# A Global Compendium of Nature-based Solutions in Small-Medium Islands

**DOI:** 10.1038/s41597-025-06476-6

**Published:** 2026-01-07

**Authors:** Mark D. C. Mansoldo, Elisa Serra, Erika Igondová, Aristides Moustakas, Abdullah Hüseyin Dönmez, Alexandra Tsatsou, Berre Kumuk, Christos Zoumides, Elli Tzirkalli, Emine Keleş Özgenç, Erich Wolff, Francesco Sica, Franziska Paul, George Zittis, Giuseppe Fenu, Hai-Ying Liu, Ioannis P. Kokkoris, Ioannis N. Vogiatzakis, Irene Christoforidi, Jean-José Filippi, Katerina-Shelagh Boucoyannis, Viviana Ligorini, Marilena Stamatiou, Marina Antic, Osman Kumuk, Periklis Kleitou, Paraskevi Manolaki, Peter Davids, Rocío Pineda-Martos, Savvas Zotos, Senka Ždero, Shiri Zemah-Shamir, Tanya Trenkova, Yael Shaked Mandelberg, Ziv Zemah-Shamir, Zorica Srđević, Mario V. Balzan

**Affiliations:** 1 Ecostack Innovations, 2100, KBIC, Kordin, Paola PLA 3000 Malta; 2 International Marine Centre (IMC), Oristano, Italy; 3 Department of Life and Environmental Sciences, University of Cagliari, Cagliari, Italy; 4 Natural History Museum of Crete, University of Crete, Heraklion, Crete Greece; 5 Department of Product and Systems Engineering, University of the Aegean, Syros, Greece; 6 Department of Soil Science and Ecology, Faculty of Forestry, Düzce University, Düzce, Türkiye; 7 Sanitary Engineering Laboratory, Department of Water Resources and Environmental Engineering, School of Civil Engineering, National Technical University of Athens, Athens, Greece; 8 Automotive Technologies Program, Iskenderun Vocational School of Higher Education, Iskenderun Technical University, 31200 Hatay, Türkiye; 9 Energy, Environment and Water Research Centre (EEWRC), The Cyprus Institute, Nicosia, Cyprus; 10 Faculty of Pure and Applied Sciences, Open University of Cyprus, Nicosia, Cyprus; 11 Department of Landscape Architecture, Faculty of Architecture, University of Trakya, Edirne, Türkiye; 12 Utrecht University, Utrecht, Netherlands; 13 Department of Architecture and Design, Sapienza University of Rome, Via Flaminia 359, 00196 Rome, Italy; 14 Ensphere, Hamburg, Germany; 15 Climate and Atmosphere Research Center (CARE-C), The Cyprus Institute, Nicosia, Cyprus; 16 Department of Environmental Impacts and Sustainability, NILU – The Climate and Environmental Research Institute NILU, Kjeller, Norway; 17 Department of Sustainable Agriculture, University of Patras, 30131 Agrinio, Greece; 18 Department of Soil, Plant and Food Sciences, University of Bari Aldo Moro, Bari, Italy; 19 Department of Agriculture, Hellenic Mediterranean University, 71410 Heraklion, Crete Greece; 20 Unité d’Appui et de Recherche CNRS 3514 Stella Mare, Université de Corse Pasquale Paoli, 20620 Biguglia, France; 21 School of Architecture, National Technical University of Athens, Athens, Greece; 22 Institute of Genetic Resources University of Banja Luka, Banja Luka, Bosnia and Herzegovina; 23 Department of Electronical and Automation, Iskenderun Vocational School of Higher Education, Iskenderun Technical University, 31200 Hatay, Türkiye; 24 Marine & Environmental Research (MER) Lab, 202 Amathountos Avenue, Marina Gardens, Block B, Off. 13-14, Limassol, Cyprus; 25 Department of Biology, Aarhus University, Ole Worms Allé 1, 8000 Aarhus C, Denmark; 26 Department of Spatial Planning, TU Dortmund University, Dortmund, Germany; 27 Departamento de Ingeniería Aeroespacial y Mecánica de Fluidos, Escuela Técnica Superior de Ingeniería Agronómica, Universidad de Sevilla, Ctra. de Utrera, km. 1, 41005 Sevilla, Spain; 28 Faculty of Agriculture, Department of Water Management, University of Novi Sad, Trg Dositeja Obradoviča 8, 21101 Novi Sad, Serbia; 29 School of Sustainability, Reichman University, Herzliya, Israel; 30 National Institute of Geophysics, Geodesy and Geography – Bulgarian Academy of Sciences, Sofia, Bulgaria; 31 Morris Kahn Marine Research Station, Leon H. Charney School of Marine Sciences, University of Haifa, Haifa, Israel; 32 Institute of Applied Sciences, Malta College of Arts, Science and Technology (MCAST), Paola, PLA 9032 Malta

**Keywords:** Ecosystem services, Geography

## Abstract

Small and medium-sized islands (SMI) combine high ecological value with limited resources and vulnerability to climatic and environmental risks. Nature-based solutions (NbS) can contribute to addressing some of these challenges, but studies on the uptake and effectiveness of NbS in SMI remain scattered, with few systematic syntheses. Here, we introduce the SMI-NbS compendium, a comprehensive and open-access dataset compiling 280 NbS case studies implemented across SMI worldwide, developed through a systematic review of published and grey literature. Each SMI-NbS case study includes information on the location, NbS category, ecosystem types, societal challenges addressed, associated co-benefits, and links to the United Nations’ Sustainable Development Goals (SDGs). The SMI-NbS compendium provides practical information on NbS implementation and identifies current research trends and gaps, such as the dominance of ecological and climate-focused NbS, with limited integration of other socio-economic challenges, thereby supporting further research and enabling knowledge exchange across the science-policy-practice interface to inform sustainable development pathways in SMI.

## Background & Summary

Small and medium-sized islands (SMI) are widely recognised as ecologically significant yet highly vulnerable regions. They have high ecological value, provide critical ecosystem services to human communities, and are considered vital biodiversity conservation hotspots^1–3^. However, their unique and intrinsic characteristics - such as the limited land area, peripheral or isolated location, and economic dependence on climate-sensitive ecosystems - make SMI especially susceptible both to climatic and non-climatic stressors^4–7^. Rising sea levels and warming temperatures, changes in precipitation patterns and the intensification of extreme events, together with unsustainable development practices, threaten not only their natural resources but also the livelihoods and well-being of the communities that depend on them^8,9^. Here, SMI are defined as islands with a surface area of <20,000 km^2^ and a human population of <1,000,000, treating each island separately within archipelagos. Hence, under this definition, islands such as Cyprus, each of the Galápagos Islands and Barbados qualify as SMI, whereas Sumatra and Sicily are excluded.

The importance of SMI is underscored by international frameworks, such as the 1992 Earth Summit’s Agenda 21, which recognise small islands as a special case for sustainable development. Later, the Small Island Developing States Accelerated Modalities of Action (SAMOA Pathway) recognised the extraordinary biological richness, ecological value, and shared threats of small island nations, and called for efforts to conserve their biodiversity, ensure its sustainable use, and promote the fair and equitable sharing of the benefits arising from it. The UN’s Sustainable Development Goals (SDGs) specifically highlight the challenges faced by small island developing states in climate resilience, marine resource management, and sustainable economic development. Indeed, they are highly susceptible to exogenous pressures, which can be disproportionately more destructive than in larger states^10^. Other SDGs, particularly those linked to disaster risk reduction, sustainable tourism, and water security, align closely with the structural challenges limiting sustainable growth in these islands.

In this context, nature-based solutions (NbS) have gained prominence as viable global, regional and local strategies to address a range of environmental, economic and societal challenges^11^, identified in climate change adaptation and mitigation, disaster risk reduction, water and food security, human health and well-being, economic and social development, as well as in reversing biodiversity loss^9,11^. Defined as “*actions that protect, sustainably manage, and restore ecosystems whilst simultaneously addressing societal challenges*”^12^, NbS leverage ecosystem functions to enhance resilience, economic sustainability, and community well-being.

**Fig. 1:**
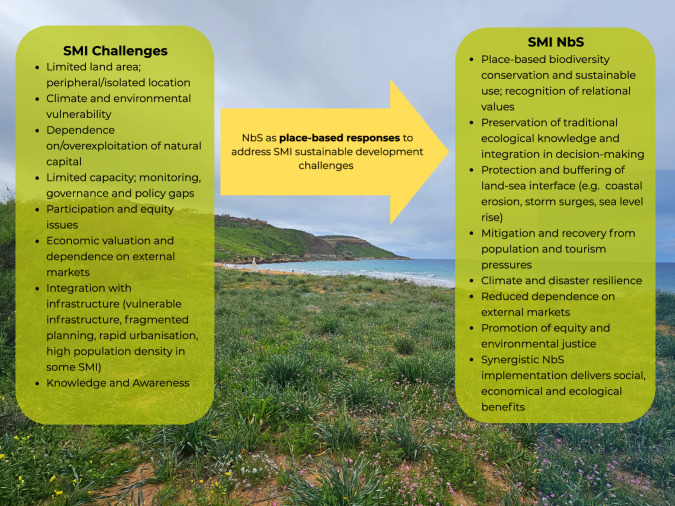
Nature-based solutions (NbS) as responses to address key sustainability challenges on small and medium-sized islands (SMI).

NbS can contribute to addressing some of the challenges faced by SMI that hinder sustainable development^13^ (Fig. 1). Such challenges are expected to be more strongly felt in SMI^13,14^, where achieving sustainable development is hindered by poorly defined socio-economic and environmental policy objectives, limited monitoring, and low availability of good quality data at the local scale, as well as by the lack of horizontal integration of environmental objectives in decision-support systems and policy-making^8,9,15^. Despite the multitude of literature reviews published on the topic of NbS^16,17^, to the best of our knowledge, a comprehensive state-of-the-art database on NbS implementation in SMI is lacking, and empirical studies on the effectiveness of NbS in SMI remain scattered with few systematic syntheses, thus underscoring the need for further research and data-driven analysis. Key knowledge and practice gaps related to the understanding of NbS principles and cost-effectiveness, and their adaptation to the local social-ecological-technological conditions, continue to limit the uptake of NbS globally and regionally^18^.

To address this gap, we have developed an open-access and easily usable global compendium of SMI-NbS. The dataset includes case studies extracted from a large number of peer-reviewed scientific papers, as well as from the grey literature. The SMI-NbS compendium described here provides a comprehensive overview of how NbS are being implemented in SMI contexts, focusing on their key features. It includes information about the country, location within FAO Major Fishing Areas^19^, human population, island size, project status, project duration, ecosystem type, NbS category, key actors and stakeholders, funding sources, SDGs addressed, societal challenges and co-benefits targeted, in addition to the methodology used to create, design, establish, and monitor NbS in the examined case studies. References, DOI and hyperlinks to the specific projects or articles have also been provided in the supplementary information.

## Methods

### Systematic analysis of peer-reviewed studies

The systematic scientific literature review was conducted in August 2024 with the Scopus multidisciplinary database of peer-reviewed and industry publications, using search keywords and Boolean operators applied to the “title”, “abstract”, and “keywords” of the publications. In developing the search protocol, we considered varying terminology under the NbS umbrella, accounting for terms that are sometimes synonymously used, or which overlap substantially with the NbS concept, as described by Dunlop *et al*.^20^. The proposed keywords were combined using the Boolean operator ‘OR’, such that the database would retrieve any publication that included any of the search terms in the title (TITLE), abstract (ABS) or author keywords (KEY):

TITLE-ABS-KEY = (“green infrastructure” OR “nature-based solutions” OR “ecological restoration” OR “ecosystem restoration” OR “ecosystem-based restoration” OR “ecosystem-based approach” OR “engineering with nature” OR “ecosystem-based adaptation” OR “natural infrastructure” OR “working with nature” OR “working with natural processes” OR “soft engineering”) AND “island*”

Literature screening followed the PRISMA method^21^. Explicit inclusion and exclusion criteria for the screening phases were defined prior to the analysis of abstracts and full texts. Articles were screened in two stages: (i) based on their title and abstract, and (ii) their full text. The title and abstract were screened for each article, and those considered potentially relevant based on title and abstract were included in the full-text review stage. In both stages, the studies were included only if they matched all of the following inclusion criteria:

- Published in English;
- Based on empirical research, i.e., not review articles or theoretical/non-empirical studies;
- Conducted on a single small and/or medium-sized island and/or its surrounding coastal area, with each island considered separately if part of an archipelago. SMI are defined here as islands having a surface area of <20,000 km^2^ and a human population of <1,000,000 inhabitants;
- Included at least one case study of NbS implementation. Key features of SMI-NbS case studies include the following:
  - Comprising or supported by nature;
  - Addressing key societal challenges associated with climate change, natural disasters, social and economic development, human health and well-being, water and food security, ecosystem degradation and biodiversity loss^11,12^;
  - Leading to benefits for biodiversity and/or human well-being.

### Case studies of NbS from the grey literature

In addition to the peer-reviewed literature, NbS case studies from the grey literature were also considered due to the numerous NbS repositories present online, which may capture additional projects beyond academic sources. Recent efforts have focused on sharing practical experiences of NbS implementation across regions in order to demonstrate benefits and share solutions and strategies to overcome barriers to NbS implementation^18,22^. This approach has been adopted by several European-funded projects and global initiatives, offering an opportunity to share experiences relating to SMI-NbS across different regions. Therefore, we have considered eight repositories of NbS, listed in Table 1, which were consulted for case studies of SMI-NbS. Appropriate case studies were then extracted from these repositories through filtering by geographic context, keywords, or manual searches. The inclusion criteria and the information obtained mirrored those mentioned above for the peer-reviewed literature.

**Table 1:** Overview of NbS repositories considered for data collection.

Repository	Objective	Scope	Key Features	Managing Organisations
PANORAMA^305^	Shares and promotes replicable NbS for cross-sectoral learning and innovation.	Global	Case studies from diverse sectors; searchable by theme and region.	Consortium of 12 organisations incl. IUCN, GIZ, UNDP, UNEP, World Bank.
Oppla^306^	Serves as a knowledge hub for NbS, natural capital, and ecosystem services.	Primarily EU; global content included	Case studies, tools, and collaborative platform.	Oppla consortium, EU-funded.
Urban Nature Atlas^307^	Catalogues urban NbS for resilience and sustainability.	Europe; global cases	>1,000 urban NbS examples with project data and filters.	NATURVATION project, CEU.
Nature4Climate^308^	Scales up NbS to address climate change and promote nature-positive action.	Global	>200 projects, tools and communication resources.	Global coalition of environmental organisations.
NetworkNature^309^	Facilitates collaboration and increases uptake of NbS across sectors.	Europe; global reach	Repository of EU-funded projects, knowledge gaps and case studies.	EU Horizon 2020-funded consortium.
Kiwa Initiative^310^	Supports climate adaptation through NbS in Pacific Island communities.	Pacific region	Funded projects showcasing resilience-building through NbS.	AFD and partners (EU, Australia, Canada, etc.).
Equator Initiative^311^	Promotes grassroots solutions contributing to SDGs via NbS.	Global	Local/indigenous-led case studies searchable by theme.	UNDP-led partnership with civil society and governments.
WOCAT^312^	Shares sustainable land management (SLM) practices.	Global	Database of 2,480 SLM practices from 136 countries.	WOCAT Secretariat, University of Bern.

### Data alignment

**Fig. 2:**
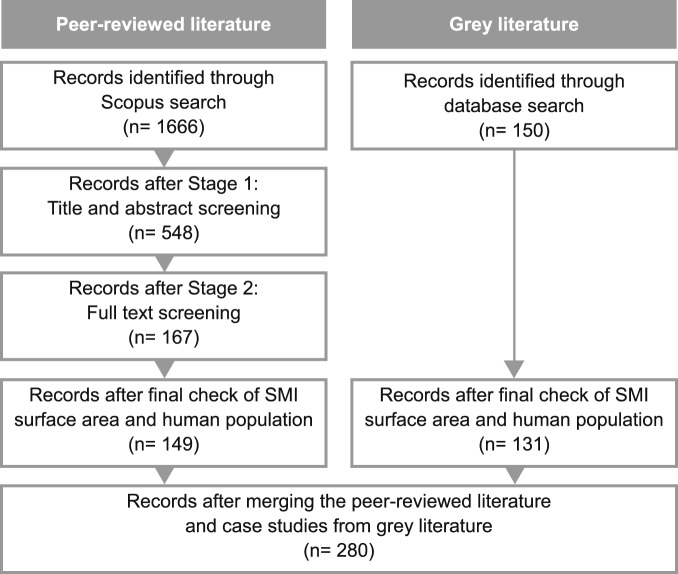
Methodological flow chart for the identification of NbS case studies in SMI.

Following quality assurance and technical validation (refer to Technical Validation), the data collected from the published scientific and grey literature were merged into a single database for data screening, including alignment of all geographic terminology and aggregation according to the FAO Major Fishing Areas^19^ (Fig. 2).

### Topic modelling

Latent Dirichlet Allocation (LDA) was applied to the combined project titles and descriptions, using the R package *‘topicmodels’*. The text was pre-processed (lowercasing, punctuation, stopword and number removal, lemmatisation), with a custom stopword list that was developed iteratively to exclude generic and domain-irrelevant terms. Topic models were estimated through Gibbs sampling (1,000 burn-in; 2,000 sampling iterations; thinning interval = 100). The number of topics (k) was determined by testing on a range of candidate values (5–20), and the final selected model was based on topic coherence scores^23^, which evaluates the semantic consistency of the top words in each topic, based on their co-occurrence patterns in the corpus^24^. For each candidate k, the model fit was repeated 5 times with fixed seeds and computed mean coherence across topics. The smallest k within one standard deviation of the maximum mean coherence was retained, leading to the identification of five topics that provided the most parsimonious data representation.

**Fig. 3:**
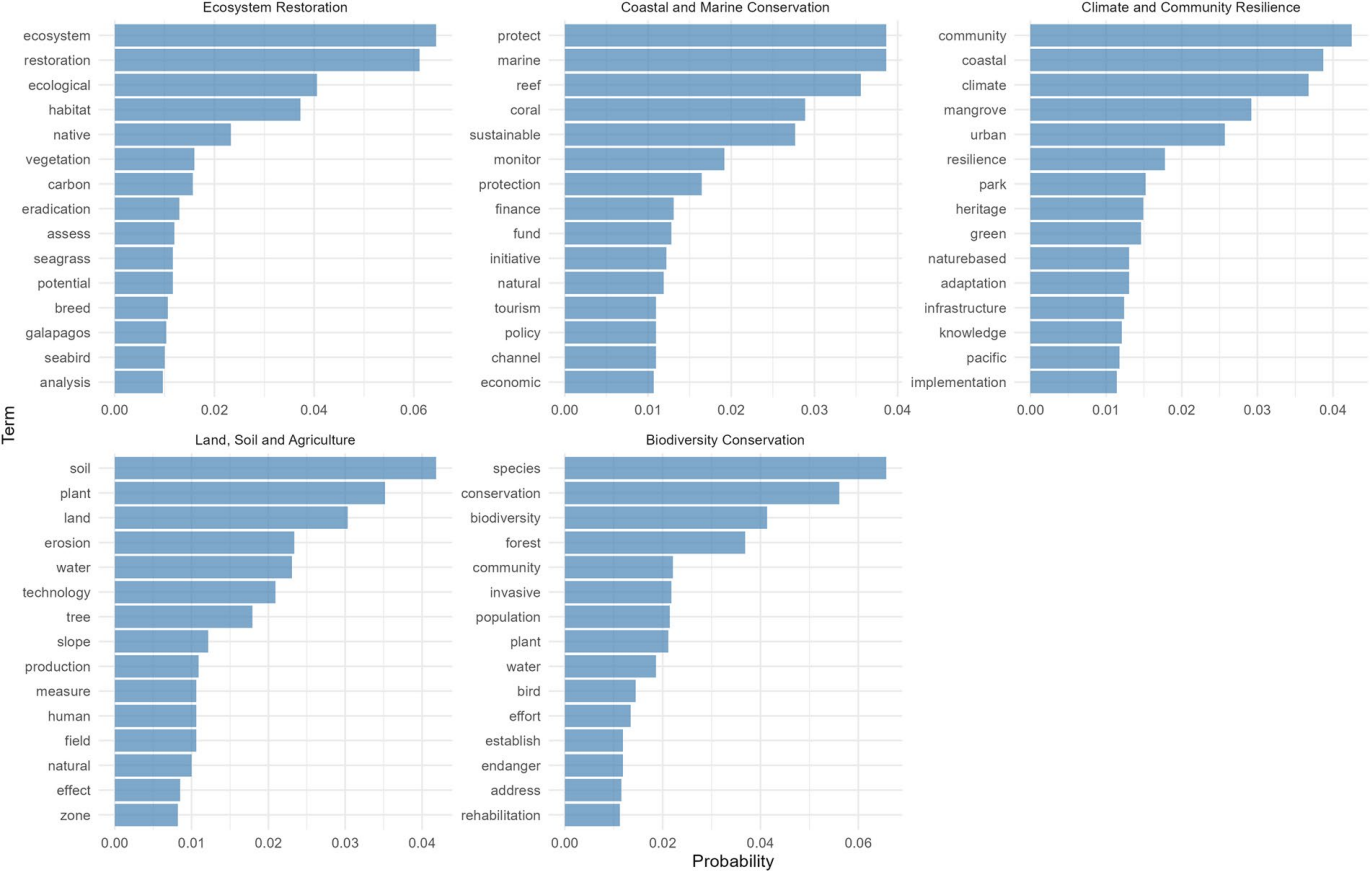
Top terms and their associated probabilities for each of the 5 topics identified through Latent Dirichlet Allocation (LDA) using Gibbs sampling. The topics reflect different thematic clusters across NbS case studies.

Topic interpretation was based on the 15 highest-probability terms per topic (Fig. 3), and the full posterior topic probability distribution for each project was recorded, allowing for weighting projects across multiple topics. Each case study was also assigned to a specific topic based on the maximum posterior topic probability for the document. The resulting five topics were interpreted as follows:

- **Ecosystem Restoration**: focusing on ecological restoration, habitat and vegetation management, carbon sequestration, eradication of invasive species, and ecological assessments. Seagrass and coastal habitats are identified, as well as seabird communities and ecosystems in the Galápagos Islands.
- **Coastal and Marine Conservation**: focusing on marine and reef ecosystems, integrating finance, tourism, and policy initiatives that support coastal and marine conservation and the blue economy.
- **Climate and Community Resilience:** focusing on climate adaptation and community-based resilience across coastal, urban, and island contexts, including mangrove restoration, green areas, heritage spaces, and parks, and the implementation of nature-based solutions and infrastructure.
- **Land, Soil and Agriculture**: focusing on soil conservation, erosion control, and land management, through vegetation and water management, as well as technologies that support agricultural practices.
- **Biodiversity Conservation**: focusing on biodiversity conservation and management of species, communities and ecosystems, including through invasive species control, conservation of endangered species and rehabilitation of ecosystems.

## Data Records

The SMI-NbS compendium and all supplementary information can be accessed on Figshare by following the link (10.6084/m9.figshare.29376149)^25^, named as follows:

- *SMI-NbS compendium* in Microsoft Excel format.
- *Overview of the SMI-NbS compendium structure*: An overview of the SMI-NbS compendium structure, describing all variables and data types, provided in Microsoft Word format.
- *Data sources*: A complete list of references, hyperlinks, and DOI links for each of the case studies listed in the SMI-NbS compendium, provided in Microsoft Excel format.

**Fig. 4:**
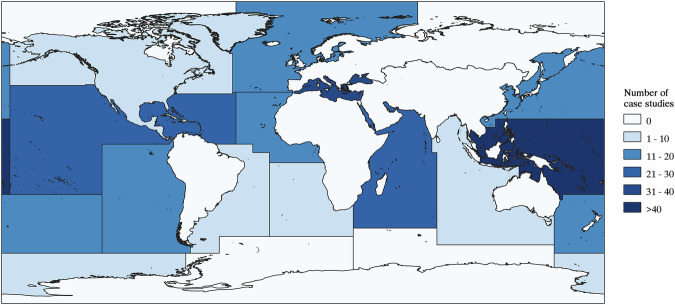
Map of NbS case studies implemented across FAO Major Fishing Areas^19^.

Within the SMI-NbS compendium, each row contains a single NbS case study, and in total the SMI-NbS compendium comprises 280 records^26–304^ across 17 FAO Major Fishing Areas^19^ (Fig. 4). General information in the compendium includes the case study ID, the associated project or peer-reviewed publication title, a short project description, the country and island of implementation, and the location within the FAO Major Fishing Areas^19^. The SMI-NbS compendium also provides an overview of how NbS are implemented in small and medium-sized insular contexts, with a focus on key features and societal challenges addressed. A full description of all fields is provided in the file named *Overview of the SMI-NbS compendium structure*, accompanied by a full list of references for each case study in the file named *Data sources*, both found on the Figshare repository alongside the SMI-NbS compendium.

## Data Overview

**Fig. 5:**
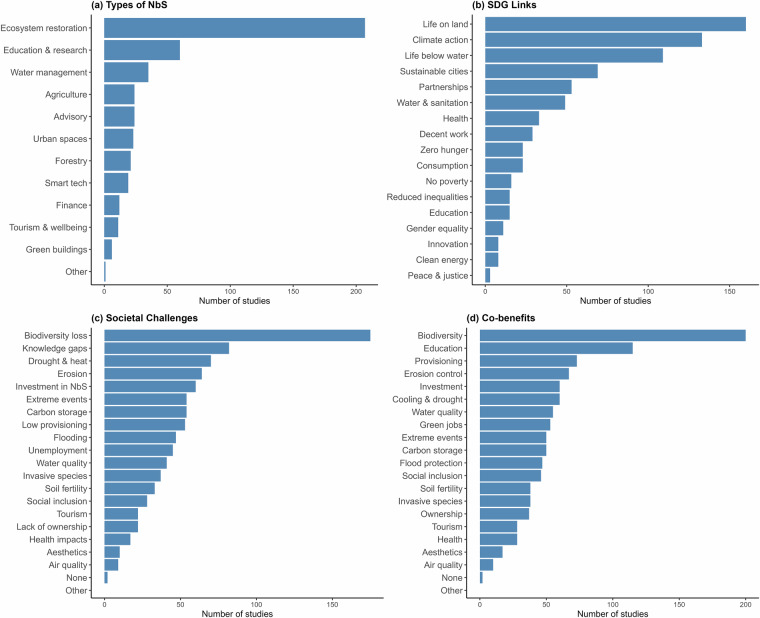
(**a**) Types of NbS implemented across the selected studies; (**b**) Number of studies addressing each SDG; (**c**) Societal challenges targeted by SMI-NbS interventions; (**d**) Reported co-benefits of NbS. Bars indicate the number of studies referring to each category, as indicated on the x-axis of the graphs.

Most studies (74%) reported the implementation of NbS for ecosystem management and restoration (Fig. 5a), and the interventions were most commonly associated with SDG15 (Life on land), SDG13 (Climate action), and SDG14 (Life below water) (Fig. 5b), reflecting a strong emphasis on biodiversity and climate mitigation and adaptation outcomes. SDGs primarily linked to the social and economic pillars of sustainable development were less prominently mentioned (Fig. 5b). The most frequently addressed societal challenge was biodiversity loss, followed by limited knowledge, climate adaptation (drought and heat), and erosion (Fig. 5c). Reported co-benefits most often include enhanced biodiversity, educational value, and provisioning benefits (e.g. food, water), with a notable presence of climate mitigation and adaptation, and socio-economic benefits (Fig. 5d). These results highlight the need to mainstream the social, cultural and economic dimensions of NbS, and to improve consideration of the instrumental, intrinsic and relational values of nature in achieving effective, equitable and place-based outcomes that contribute to the sustainability of SMI.

**Fig. 6:**
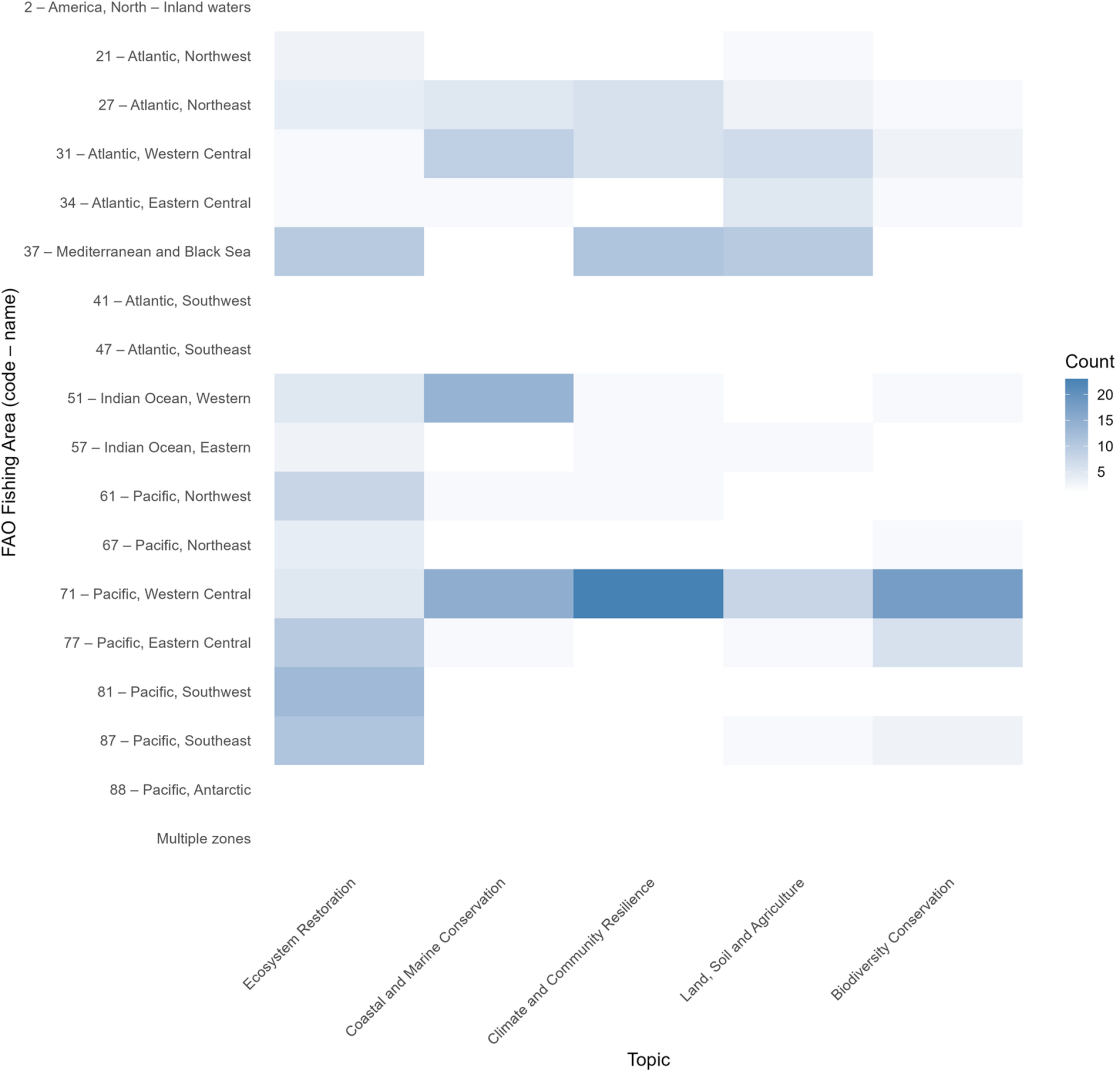
Heatmap showing the distribution of dominant LDA-derived topics across FAO Major Fishing Areas^19^. Each cell represents the number of case studies assigned to a given topic (x-axis) within a specific FAO zone (y-axis), with intensity reflecting topic frequency. Multi-zone entries – i.e. projects associated with more than one FAO area – are grouped under ‘Multiple zones’.

Topic modelling across FAO Major Fishing Areas^19^ showed clear regional patterns in SMI-NbS implementation (Fig. 6). *Ecosystem restoration* was most prevalent in the Southwestern (Area 81), Southeastern (Area 87), and Eastern Central (Area 77) areas of the Pacific, reflecting restoration and habitat management activities across Pacific Island ecosystems, as well as in the Mediterranean and Black Sea (Area 37). *Coastal and marine conservation* was concentrated in the Western Central Pacific (Area 71) and the Western Indian Ocean (Area 51), with several cases also documented from the Western Central Atlantic (Area 31). *Climate and community resilience* emerged strongly in the Western Central Pacific (Area 71) and the Mediterranean and Black Sea (Area 37). *Land, soil and agriculture* were more prevalent in the Mediterranean and Black Sea (Area 37) but were also well represented in the Western Central Pacific (Area 71) and Central Atlantic (Areas 31 and 34). *Biodiversity conservation* case studies were most predominantly associated with the Western Central Pacific (Area 71).

Overall, the Western Central Pacific (Area 71) was the most thematically diverse FAO Major Fishing Area, with NbS projects represented across all five topics. NbS case studies in this region were broadly distributed across several SMI, including Viti Levu, Palau, Efate, Fiji, Vanuatu, French Polynesia, the Marshall Islands, Tonga, and New Caledonia, with most islands represented by only a few case studies. This contrasts with the Mediterranean and Black Sea (Area 37), where case studies were more geographically clustered and concentrated in the Balearic Islands, Crete and, to a lesser extent, the Maltese Islands.

Several other regions were also dominated by fewer topics, reflecting a more specialised thematic profile of NbS implementation and highlighting how regional contexts shape priorities and approaches to NbS in SMI. Given the fragmentation of SMI NbS case studies, these results emphasise the importance of fostering collaboration between islands and regions, where regional and global capacity-building, collaboration and financing initiatives on island biodiversity, climate action, and sustainability, can promote the exchange of knowledge and experiences, addressing evidence and practice gaps^13^, as well as supporting the scaling of effective NbS to shared challenges faced by SMI.

## Technical Validation

To check the level of agreement among the co-authors, a quality assurance process was carried out on approximately 10% (n = 56) of the peer-reviewed articles that had undergone the second stage of review (n = 548). Of the cross-validated articles, there was 71% agreement among reviewers, 13% of uncertainty (involving ‘maybe include’), and 16% conflict. Any conflicts were resolved by exploring the justifications of the respective reviewers and excluding or including the papers where appropriate.

One final screening of all peer-reviewed and grey literature was then conducted to ensure that all studies which referred to SMI NbS were included in the final compendium and any not deemed relevant were removed accordingly. The screening phases of the review, along with the number of publications and case studies excluded at each stage, are shown in Fig. 2.

## Data Usage

The SMI-NbS compendium addresses the goal of sharing practical, local and place-based experiences of NbS implementation across regions^22^ and highlights their multifunctionality. It has been designed as a ready-to-use knowledge resource for users working on NbS in SMI contexts. For example, the compendium could be used by scholars to further explore emerging gaps and trends relating to NbS in SMI, such as variations in NbS types, geographic distribution and funding patterns. Decisionmakers and practitioners working at the local scale may draw upon the case studies to examine how NbS contribute to different policy objectives. The SMI-NbS compendium can additionally support the design of regional or global initiatives by facilitating knowledge exchange and learning across islands, archipelagos and regions, thus promoting the scaling up of existing success stories. Furthermore, the compendium provides information on the use of different NbS types according to island ecosystems, and in each case the societal challenges addressed, the stakeholders engaged, and the co-creation processes adopted, in turn highlighting the social, economic and environmental benefits generated.

For practical use, the compendium is openly available in a structured format that enables both exploratory consultation and systematic analysis. In the file *Data sources*, each case study is listed alongside hyperlinks or DOIs wherever available, allowing users to directly access project descriptions and primary sources. The coding system facilitates the integration of the SMI-NbS compendium with other analytical tools, thus enabling future users to conduct advanced quantitative or qualitative assessments, and comparative studies across regions.

## Data Availability

The SMI-NbS compendium and all supplementary information can be openly accessed via Figshare (10.6084/m9.figshare.29376149)^25^.

## Code availability

All codes used for data analysis and visualisation were made through existing libraries using the R version 4.3.3 in RStudio. The R script is available in the GitHub repository at https://github.com/EcoINN/SMI-NBS. The software QGIS (v.3.40) was used to render the map in Fig. 4.

## References

[1] Fenu, G. *et al*. A Common Approach to the Conservation of Threatened Island Vascular Plants: First Results in the Mediterranean Basin. *Diversity* **12**, 157 (2020).

[2] Vogiatzakis, I. *et al*. Enhancing Small-Medium IsLands resilience by securing the sustainability of Ecosystem Services: the SMILES Cost Action. *RIO* **9**, e116061 (2023).

[3] Hilmi, N. *et al*. Resilience of Small Islands: Unveiling Nature-Based Solutions for Sustainable Futures. in *Climate-Resilient Cities* (eds. Arora, A., Belaïd, F. & Lechtenberg-Kasten, S.) 257–280, 10.1007/978-3-031-73090-0_13 (Springer Nature Switzerland, Cham, 2025).

[4] *Climate Change 2007: Impacts, Adaptation and Vulnerability: Contribution of Working Group II to the Fourth Assessment Report of the Intergovernmental Panel on Climate Change*. (Cambridge University Press, Cambridge, U.K. New York, 2007).

[5] Nurse, L. A. *et al*. Small islands. in *Climate Change 2014: Impacts, Adaptation, and Vulnerability. Part B: Regional Aspects. Contribution of Working Group II to the Fifth Assessment Report of the Intergovernmental Panel on Climate Change* (eds. Barros, V.R., Field, C. B., Dokken, D. J., Mastrandrea, M. D., Mach, K. J., Bilir, T. E., Chatterjee, M., Ebi, K. L., Estrada, Y. O., Genova, R. C., Girma, B., Kissel, E. S., Levy, A. N., MacCracken, S., Mastrandrea, P. R. & White (eds.), and L. L.) 1613–1654 (Cambridge University Press, 2014).

[6] Cramer, W. *et al*. Climate change and interconnected risks to sustainable development in the Mediterranean. *Nature Clim Change* **8**, 972–980 (2018).

[7] Moustakas, A. *et al*. Climate land use and other drivers’ impacts on island ecosystem services: A global review. *Science of The Total Environment* **973**, 179147 (2025). 40112548 10.1016/j.scitotenv.2025.179147

[8] Intergovernmental Panel On Climate Change (IPCC). *Climate Change 2022 – Impacts, Adaptation and Vulnerability: Working Group II Contribution to the Sixth Assessment Report of the Intergovernmental Panel on Climate Change*, 10.1017/9781009325844 (Cambridge University Press, 2023).

[9] Zittis, G. *et al*. Insular ecosystem services in peril: a systematic review on the impacts of climate change and other drivers. *Climatic Change* **178**, 127 (2025).

[10] Bishop, M. *et al*. Towards sustained development in Small Island Developing States. *ODI working paper* 46 (2021).

[11] IUCN, International Union for Conservation of Nature. *IUCN Global Standard for Nature-Based Solutions: A User-Friendly Framework for the Verification, Design and Scaling up of NbS: First Edition*, 10.2305/IUCN.CH.2020.08.en (IUCN, International Union for Conservation of Nature, 2020).

[12] *Nature-Based Solutions to Address Global Societal Challenges*, 10.2305/IUCN.CH.2016.13.en (IUCN International Union for Conservation of Nature, 2016).

[13] Grace, M. *et al*. Priority knowledge needs for implementing nature-based solutions in the Mediterranean islands. *Environmental Science & Policy* **116**, 56–68 (2021).

[14] Balzan, M. V. *et al*. Assessing nature-based solutions uptake in a Mediterranean climate: insights from the case-study of Malta. *Nature-Based Solutions* **2**, 100029 (2022).

[15] Balzan, M. V., Potschin-Young, M. & Haines-Young, R. Island ecosystem services: insights from a literature review on case-study island ecosystem services and future prospects. *International Journal of Biodiversity Science, Ecosystem Services & Management* **14**, 71–90 (2018).

[16] Debele, S. E. *et al*. Nature-based solutions can help reduce the impact of natural hazards: A global analysis of NBS case studies. *Science of The Total Environment* **902**, 165824 (2023). 37527720 10.1016/j.scitotenv.2023.165824

[17] Lehmann, I. *et al*. Time in and for nature-based solutions. No quick fix solutions for complex ecological and social processes. *Nature-Based Solutions* **7**, 100219 (2025).

[18] Castellar, J. A. C. *et al*. What does it take to renature cities? An expert-based analysis of barriers and strategies for the implementation of nature-based solutions. *Journal of Environmental Management* **354**, 120385 (2024). 38382435 10.1016/j.jenvman.2024.120385

[19] Food and Agriculture Organization of the United Nations. FAO Major Fishing Areas. *FAO Major Fishing Areas* https://www.fao.org/fishery/en/area/search.

[20] Dunlop, T. *et al*. The evolution and future of research on Nature-based Solutions to address societal challenges. *Commun Earth Environ* **5**, 132 (2024).

[21] Page, M. J. *et al*. The PRISMA 2020 statement: an updated guideline for reporting systematic reviews. *BMJ* n71, 10.1136/bmj.n71 (2021). 10.1136/bmj.n71 PMC8005924 33782057

[22] Faivre, N., Fritz, M., Freitas, T., De Boissezon, B. & Vandewoestijne, S. Nature-Based Solutions in the EU: Innovating with nature to address social, economic and environmental challenges. *Environmental Research* **159**, 509–518 (2017). 28886502 10.1016/j.envres.2017.08.032

[23] Jones, T. _textmineR: Functions for Text Mining and Topic Modeling_ (R package version 3.0.5).

[24] Mimno, D., Wallach, H., Talley, E., Leenders, M. & McCallum, A. Optimizing semantic coherence in topic models. in *Proceedings of the 2011 conference on empirical methods in natural language processing* 262–272 (2011).

[25] Mansoldo, M. D. C., Balzan, M. V., Serra, E., Igondová, E. & Moustakas, A. The SMI-NBS Compendium: A Global Compendium of Nature-based Solutions in Small-Medium Islands. *Figshare*10.6084/m9.figshare.29376149 (2025). 10.1038/s41597-025-06476-6 PMC12873128 41501094

[26] Algar, D., Morris, K., Asher, J. & Cowen, S. Dirk Hartog Island ‘Return to 1616’ Project – The first six years (2014 to 2019). *Eco Management Restoration* **21**, 173–183 (2020).

[27] Ammondt, S. A., Litton, C. M., Ellsworth, L. M. & Leary, J. K. Restoration of native plant communities in a Hawaiian dry lowland ecosystem dominated by the invasive grass *M**egathyrsus maximus*. *Applied Vegetation Science* **16**, 29–39 (2013).

[28] Anderson, R. M. & Lambert, A. M. Endangered Butterflies and their Non-Native Host Plants: Examining Shifting Values of Belonging in Restoration. *Case Studies in the Environment* **3**, 1–9 (2019).

[29] Arévalo, J. R., Delgado, J. D. & Fernández-Palacios, J. M. Regeneration of potential laurel forest under a native canopy vs. exotic canopy, Tenerife (Canary Islands). *For. syst.* **20**, 255–265 (2011).

[30] Arévalo, J. R. & Fernández-Palacios, J. M. From Pine Plantations to Natural Stands. Ecological Restoration of a *Pinus canariensis* Sweet, ex Spreng forest. *Plant Ecol* **181**, 217–226 (2005).

[31] Armada, N., White, A. T. & Christie, P. Managing Fisheries Resources in Danajon Bank, Bohol, Philippines: An Ecosystem-Based Approach. *Coastal Management* **37**, 308–330 (2009).

[32] Audet, P. *et al*. Structural development of vegetation on rehabilitated North Stradbroke Island: Above/belowground feedback may facilitate alternative ecological outcomes. *Ecol Process* **2**, 20 (2013).

[33] Bade, D. Issues and Tensions in Island Heritage Management: A Case Study of Motuihe Island, New Zealand. *ISJ* **5**, 25–42 (2010).

[34] Balzan, M. V., Caruana, J. & Zammit, A. Assessing the capacity and flow of ecosystem services in multifunctional landscapes: Evidence of a rural-urban gradient in a Mediterranean small island state. *Land Use Policy* **75**, 711–725 (2018).

[35] Berkowitz, B. & Medley, K. Home Gardenscapes as Sustainable Landscape Management on St. Eustatius, Dutch Caribbean. *Sustainability* **9**, 1310 (2017).

[36] Braschi, J., Torres, A., Fadda, S., Buisson, E. & Ponel, P. Beetle assemblage dynamics after invasive ice plant (*Carpobrotus*) removal on a small Mediterranean island. *Restoration Ecology* **29**, e13387 (2021).

[37] Brathwaite, A., Clua, E., Roach, R. & Pascal, N. Coral reef restoration for coastal protection: Crafting technical and financial solutions. *Journal of Environmental Management* **310**, 114718 (2022). 35192980 10.1016/j.jenvman.2022.114718

[38] Bremer, L. L. *et al*. Contributions of native forest protection to local water supplies in East Maui. *Science of The Total Environment* **688**, 1422–1432 (2019). 31726570 10.1016/j.scitotenv.2019.06.220

[39] Buckwell, A. *et al*. Social benefit cost analysis of ecosystem-based climate change adaptations: a community-level case study in Tanna Island, Vanuatu. *Climate and Development* **12**, 495–510 (2020).

[40] Buddenhagen, C. E. & Tye, A. Lessons from successful plant eradications in Galapagos: commitment is crucial. *Biol Invasions* **17**, 2893–2912 (2015).

[41] Bull, L. S. Factors Influencing Little Penguin *Eudyptula minor* Egg Success on Matiu-Somes Island, New Zealand. *Emu - Austral Ornithology* **100**, 199–204 (2000).

[42] Burgess, B. T., Irvine, R. L. & Russello, M. A. Population genomics of Sitka black-tailed deer supports invasive species management and ecological restoration on islands. *Commun Biol* **5**, 223 (2022). 35273319 10.1038/s42003-022-03159-5 PMC8913846

[43] Camacho, I., Macías-de-la-Rosa, Á., Camacho, R., Grinn-Gofroń, A. & Cariñanos, P. The allergenic potential of green urban areas in the Macaronesian islands: The case of Funchal City (Madeira). *Urban Climate* **54**, 101866 (2024).

[44] Carrion, V., Donlan, C. J., Campbell, K. J., Lavoie, C. & Cruz, F. Archipelago-Wide Island Restoration in the Galápagos Islands: Reducing Costs of Invasive Mammal Eradication Programs and Reinvasion Risk. *PLoS ONE* **6**, e18835 (2011). 21589656 10.1371/journal.pone.0018835 PMC3092746

[45] Caselle, J. E., Rassweiler, A., Hamilton, S. L. & Warner, R. R. Recovery trajectories of kelp forest animals are rapid yet spatially variable across a network of temperate marine protected areas. *Sci Rep* **5**, 14102 (2015). 26373803 10.1038/srep14102 PMC4642697

[46] Cevallos, D. & Jaramillo Díaz, P. Assessing Water-Saving Technologies and the Impact of Giant Tortoise Herbivory on the Restoration of *Opuntia megasperma* var. *orientalis* on Española Island—Galapagos. *Water* **16**, 369 (2024).

[47] Chee, S. Y. *et al*. Drill-Cored Artificial Rock Pools Can Promote Biodiversity and Enhance Community Structure on Coastal Rock Revetments at Reclaimed Coastlines of Penang, Malaysia. *Tropical Conservation Science* **13**, 1940082920951912 (2020).

[48] Chee, S. Y. *et al*. Habitat Complexity Affects the Structure but Not the Diversity of Sessile Communities on Tropical Coastal Infrastructure. *Front. Ecol. Evol.* **9**, 673227 (2021).

[49] Chen, W., Staneva, J., Jacob, B., Sánchez-Artús, X. & Wurpts, A. What-if nature-based storm buffers on mitigating coastal erosion. *Science of The Total Environment* **928**, 172247 (2024). 38599407 10.1016/j.scitotenv.2024.172247

[50] Chenot, J., Affre, L., Passetti, A. & Buisson, E. Consequences of iceplant (*Carpobrotus*) invasion on the vegetation and seed bank structure on a Mediterranean island: response elements for their local eradication. *Acta Botanica Gallica* **161**, 301–308 (2014).

[51] Chowdhury, M. S. N. *et al*. Oyster breakwater reefs promote adjacent mudflat stability and salt marsh growth in a monsoon dominated subtropical coast. *Sci Rep* **9**, 8549 (2019). 31189886 10.1038/s41598-019-44925-6 PMC6561949

[52] Clifford, M. J., Ali, S. H. & Matsubae, K. Mining, land restoration and sustainable development in isolated islands: An industrial ecology perspective on extractive transitions on Nauru. *Ambio* **48**, 397–408 (2019). 30076524 10.1007/s13280-018-1075-2 PMC6411803

[53] Copson, G. & Whinam, J. Review of ecological restoration programme on subantarctic Macquarie Island: Pest management progress and future directions. *Eco Management Restoration* **2**, 129–138 (2001).

[54] Costa, A. *et al*. Impacts of invasive ants on pollination of native plants are similar in invaded and restored plant communities. *Global Ecology and Conservation* **42**, e02413 (2023).

[55] Crameri, N. J. & Ellison, J. C. Atoll Mangrove Progradation Patterns: Analysis from Jaluit in the Marshall Islands. *Estuaries and Coasts* **47**, 935–948 (2024).

[56] Currie, D. *et al*. Conservation options for the Critically Endangered Seychelles Black Paradise-flycatcher *Terpsiphone corvina*. *Bird Conservation International* **13**, 97–114 (2003).

[57] Daehler, C. C. & Goergen, E. M. Experimental Restoration of an Indigenous Hawaiian Grassland after Invasion by Buffel Grass (*Cenchrus ciliaris*). *Restoration Ecology* **13**, 380–389 (2005).

[58] Davis, J. *et al*. Beneficial use of sediments to restore a Chesapeake Bay marsh island. *Front. Sustain.* **5**, 1359721 (2024).

[59] De Jong, B., Keijsers, J. G., Riksen, M. J., Krol, J. & Slim, P. A. Soft Engineering vs. a Dynamic Approach in Coastal Dune Management: A Case Study on the North Sea Barrier Island of Ameland, The Netherlands. *Journal of Coastal Research* **30**, 670 (2014).

[60] Donato, D. C., Kauffman, J. B., Mackenzie, R. A., Ainsworth, A. & Pfleeger, A. Z. Whole-island carbon stocks in the tropical Pacific: Implications for mangrove conservation and upland restoration. *Journal of Environmental Management* **97**, 89–96 (2012). 22325586 10.1016/j.jenvman.2011.12.004

[61] Donlan, C. J., Croll, D. A. & Tershy, B. R. Islands, Exotic Herbivores, and Invasive Plants: Their Roles in Coastal California Restoration. *Restoration Ecology* **11**, 524–530 (2003).

[62] Duffy, D. C. Changing Seabird Management in Hawai’i: from Exploitation through Management to Restoration. *Waterbirds* **33**, 193–207 (2010).

[63] East, K. T., East, M. R. & Daugherty, C. H. Ecological restoration and habitat relationships of reptiles on Stephens Island, New Zealand. *New Zealand Journal of Zoology* **22**, 249–261 (1995).

[64] Edgar, G. J. *et al*. Bias in evaluating the effects of marine protected areas: the importance of baseline data for the Galapagos Marine Reserve. *Envir. Conserv.* **31**, 212–218 (2004).

[65] Eichmanns, C. & Schüttrumpf, H. Investigating Changes in Aeolian Sediment Transport at Coastal Dunes and Sand Trapping Fences: A Field Study on the German Coast. *JMSE* **8**, 1012 (2020).

[66] Elliff, C. I. & Kikuchi, R. K. P. Ecosystem services provided by coral reefs in a Southwestern Atlantic Archipelago. *Ocean & Coastal Management* **136**, 49–55 (2017).

[67] Ellison, J. C. Pacific Island Beaches: Values, Threats and Rehabilitation. in *Beach Management Tools - Concepts, Methodologies and Case Studies* (eds. Botero, C. M., Cervantes, O. & Finkl, C. W.) vol. 24 679–700 (Springer International Publishing, Cham, 2018).

[68] Equator Initiative. Amal-Crab Bay Community Resource Management Initiative. https://www.equatorinitiative.org/wp-content/uploads/2017/05/case_1348152340_EN.pdf.

[69] Equator Initiative. Arnavon Community Marine Conservation (Choiseul Island). https://www.equatorinitiative.org/wp-content/uploads/2017/05/case_1348068029-EN.pdf.

[70] Equator Initiative. Arnavon Community Marine Conservation (Santa Isabel). https://www.equatorinitiative.org/wp-content/uploads/2017/05/case_1348068029-EN.pdf.

[71] Equator Initiative. Arnavon Community Marine Conservation (Wagina Island). https://www.equatorinitiative.org/wp-content/uploads/2017/05/case_1348068029-EN.pdf.

[72] Equator Initiative. Community Marine Association of Cruzinha da Garça. https://www.old.equatorinitiative.org/images/stories/winners/27/casestudy/case_1348150348.pdf.

[73] Equator Initiative. Fiji Locally Managed Marine Area Network. https://www.equatorinitiative.org/wp-content/uploads/2017/05/case_1348160544.pdf.

[74] Equator Initiative. Loru Forest Carbon. https://www.equatorinitiative.org/2019/07/29/ser-thiac/.

[75] Equator Initiative. Mohéli Marine Park. https://www.equatorinitiative.org/wp-content/uploads/2017/05/case_1348163266.pdf.

[76] Equator Initiative. Namdrik Atoll Local Resources Committee. https://www.equatorinitiative.org/wp-content/uploads/2017/05/case_1370356453.pdf.

[77] Equator Initiative. Nguna-Pele Marine and Land Protected Area Network (Nguna Island). https://www.equatorinitiative.org/wp-content/uploads/2017/05/case_1348163605.pdf.

[78] Equator Initiative. Nguna-Pele Marine and Land Protected Area Network (Pele Island). https://www.equatorinitiative.org/wp-content/uploads/2017/05/case_1348163605.pdf.

[79] Equator Initiative. SER-THIAC. https://www.equatorinitiative.org/wp-content/uploads/2021/01/Ser-Thiac-Case-Study-English-FNL-2.pdf.

[80] Equator Initiative. Tetepare Descendants’ Association. https://www.equatorinitiative.org/wp-content/uploads/2017/05/case_1370356629.pdf.

[81] Fischer, L. K., Von Der Lippe, M. & Kowarik, I. Tree invasion in managed tropical forests facilitates endemic species. *Journal of Biogeography* **36**, 2251–2263 (2009).

[82] Fleri, J. R., Lera, S., Gerevini, A., Staver, L. & Nardin, W. Empirical observations and numerical modelling of tides, channel morphology, and vegetative effects on accretion in a restored tidal marsh. *Earth Surf Processes Landf* **44**, 2223–2235 (2019).

[83] Foran, C. M., Burks-Copes, K. A., Berkowitz, J., Corbino, J. & Suedel, B. C. Quantifying Wildlife and Navigation Benefits of a Dredging Beneficial-Use Project in the Lower Atchafalaya River: A Demonstration of Engineering with Nature®. *Integrated Environmental Assessment and Management* **14**, 759–768 (2018). 29963740 10.1002/ieam.4084

[84] Forbes, A. R. & Craig, J. L. Assessing the role of revegetation in achieving restoration goals on Tiritiri Matangi Island. *New Zealand Journal of Ecology* **37**, 343–352 (2013).

[85] Forrester, G. E. *et al*. Evaluating Methods for Transplanting Endangered Elkhorn Corals in the Virgin Islands. *Restoration Ecology* **19**, 299–306 (2011).

[86] Fraser, I. D. L. *et al*. Rotoroa Island: building a designed ecosystem for conservation education, training and visitor engagement. *International Zoo Yearbook* **51**, 175–186 (2017).

[87] Furukawa, K. Case studies for urban wetlands restoration and management in Japan. *Ocean & Coastal Management* **81**, 97–102 (2013).

[88] Galbraith, M. & Cooper, H. Tiritiri Matangi - an overview of 25 years of ecological restoration. *New Zealand Journal of Ecology* **37**, 258–260 (2013).

[89] García-Gil, A., Fontes, J. C. & Santamarta, J. C. Groundwater conditions the effectiveness of surface water diversion in the remediation of the eutrophicated volcanic lake of Furnas, Azores archipelago. *Science of The Total Environment* **837**, 155789 (2022). 35561900 10.1016/j.scitotenv.2022.155789

[90] Gardner-Gee, R., Rayner, M. & Beggs, J. R. Monitoring grey-faced petrels (*Pterodroma macroptera gouldi*) in a restoration project on Motuora Island, Hauraki Gulf. *Notornis* **55**, 184 (2008).

[91] Gilman, E. & Ellison, J. Efficacy of alternative low-cost approaches to mangrove restoration, American Samoa. *Estuaries and Coasts* **30**, 641–651 (2007).

[92] Gomes, T. C. Novel ecosystems in the restoration of cultural landscapes of Tl’chés, West Chatham Island, British Columbia, Canada. *Ecol Process* **2**, 15 (2013).

[93] Griffiths, C. J. *et al*. The Use of Extant Non-Indigenous Tortoises as a Restoration Tool to Replace Extinct Ecosystem Engineers. *Restoration Ecology* **18**, 1–7 (2010).

[94] Handel, S. N., Robinson, G. R., Parsons, W. F. J. & Mattei, J. H. Restoration of Woody Plants to Capped Landfills: Root Dynamics in an Engineered Soil. *Restoration Ecology* **5**, 178–186 (1997).

[95] Hanson, C. C. *et al*. Feral cat eradication in the presence of endemic San Nicolas Island foxes. *Biol Invasions* **17**, 977–986 (2015).

[96] Hayes, M. O., Kana, T. W. & Barwis, J. H. Soft Designs for Coastal Protection at Seabrook Island, S.C. in *Coastal Engineering 1980* 897–912, 10.1061/9780872622647.056 (American Society of Civil Engineers, Sydney, Australia, 1980).

[97] He, B., Cai, Y., Ran, W. & Jiang, H. Spatial and seasonal variations of soil salinity following vegetation restoration in coastal saline land in eastern China. *CATENA* **118**, 147–153 (2014).

[98] Heriot, S., Asher, J., Williams, M. R. & Moro, D. The eradication of ungulates (sheep and goats) from Dirk Hartog Island, Shark Bay World Heritage Area, Australia. *Biol Invasions* **21**, 1789–1805 (2019).

[99] Higgs, E. The Two‐Culture Problem: Ecological Restoration and the Integration of Knowledge. *Restoration Ecology* **13**, 159–164 (2005).

[100] Hong, H.-J., Kim, C.-K., Lee, H.-W. & Lee, W.-K. Conservation, Restoration, and Sustainable Use of Biodiversity Based on Habitat Quality Monitoring: A Case Study on Jeju Island, South Korea (1989–2019). *Land* **10**, 774 (2021).

[101] Hunter, E. A. & Gibbs, J. P. Densities of Ecological Replacement Herbivores Required to Restore Plant Communities: A Case Study of Giant Tortoises on Pinta Island, Galápagos. *Restoration Ecology* **22**, 248–256 (2014).

[102] Hunter, E. A., Gibbs, J. P., Cayot, L. J. & Tapia, W. Equivalency of Galápagos Giant Tortoises Used as Ecological Replacement Species to Restore Ecosystem Functions. *Conservation Biology* **27**, 701–709 (2013). 23530938 10.1111/cobi.12038

[103] Hunter, E. A. *et al*. Seeking compromise across competing goals in conservation translocations: The case of the ‘extinct’ Floreana Island Galapagos giant tortoise. *Journal of Applied Ecology* **57**, 136–148 (2020).

[104] Ibañez-Álvarez, M. *et al*. Satellite-Based Monitoring of Primary Production in a Mediterranean Islet Post Black Rat Eradication. *Remote Sensing* **14**, 101 (2021).

[105] Dekker, I. & Fantini, E. Nature based solution for flood control in The Netherlands. Socialising water or naturifying society? *Rassegna Italiana di Sociologia* **253**, 278, 10.1423/97800 (2020).

[106] Jäger, H. & Kowarik, I. Resilience of Native Plant Community Following Manual Control of Invasive *Cinchona pubescens* in Galápagos. *Restoration Ecology* **18**, 103–112 (2010).

[107] Jenkins, A. P., Jupiter, S. D., Qauqau, I. & Atherton, J. The importance of ecosystem‐based management for conserving aquatic migratory pathways on tropical high islands: a case study from Fiji. *Aquatic Conservation* **20**, 224–238 (2010).

[108] Kamelamela, K. L. *et al*. Kōkua aku, Kōkua mai: An Indigenous Consensus-driven and Place-based Approach to Community Led Dryland Restoration and Stewardship. *Forest Ecology and Management* **506**, 119949 (2022).

[109] Kelaher, B. P., Pappagallo, T., Litchfield, S. & Fellowes, T. E. Drone-Based Monitoring to Remotely Assess a Beach Nourishment Program on Lord Howe Island. *Drones* **7**, 600 (2023).

[110] Khalil, S. M., Knotts, C. P. & Tate, B. Restoration of Louisiana Barrier Islands: Engineering Approaches to Hazard Mitigation by Modulating Coastal Environments. in *Coastal Engineering 2006* 1951–1963, 10.1142/9789812709554_0165 (World Scientific Publishing Company, San Diego, California, USA, 2007).

[111] Khirfan, L. & El-Shayeb, H. Urban climate resilience through socio-ecological planning: a case study in Charlottetown, Prince Edward Island. *Journal of Urbanism: International Research on Placemaking and Urban Sustainability* **13**, 187–212 (2020).

[112] Kittinger, J. N., Duin, K. N. & Wilcox, B. A. Commercial fishing, conservation and compatibility in the Northwestern Hawaiian Islands. *Marine Policy* **34**, 208–217 (2010).

[113] Kiwa Initiative. Advancing NbS with Youth and Vulnerable Groups in Niue. https://kiwainitiative.org/en/projects/filters/cobenefits/biodiversity-conservation/advancing-NbS-with-youth-and-vulnerable-groups-in-niue.

[114] Kiwa Initiative. ARU KOMO / Preservation of the water resource on the Anaa atoll. https://kiwainitiative.org/en/projects/filters/cobenefits/biodiversity-conservation/aru-komo.

[115] Kiwa Initiative. Biodiversity management and conservation (Eua). https://kiwainitiative.org/en/projects/filters/cobenefits/biodiversity-conservation/youth-biodiversity-management.

[116] Kiwa Initiative. Biodiversity management and conservation (Ha’ano). https://kiwainitiative.org/en/projects/filters/cobenefits/biodiversity-conservation/youth-biodiversity-management.

[117] Kiwa Initiative. Biodiversity management and conservation (Tongatapu). https://kiwainitiative.org/en/projects/filters/cobenefits/biodiversity-conservation/youth-biodiversity-management.

[118] Kiwa Initiative. Biodiversity management and conservation (Utu Vava’u). https://kiwainitiative.org/en/projects/filters/cobenefits/biodiversity-conservation/youth-biodiversity-management.

[119] Kiwa Initiative. Food security / Invasive species management. https://kiwainitiative.org/en/projects/safeguarding-rennell-island-livelihoods-and-biodiversity-from-invasive-species-project.

[120] Kiwa Initiative. Imin ira nin gatsimor (Tree of life) / The tree of life: valuing the coconut tree and its ecological and traditional significance to the nauruan people. https://kiwainitiative.org/en/projects/filters/cobenefits/biodiversity-conservation/imin-ira-nin-gatsimor-tree-of-life.

[121] Kiwa Initiative. Management and restoration of O Le Pupu Pue National Park. https://kiwainitiative.org/en/projects/nature-based-financing-for-protected-areas-in-samoa-project.

[122] Kiwa Initiative. Mangroves restoration for livelihoods. https://kiwainitiative.org/en/projects/using-nature-based-solutions-to-protect-coastal-communities-from-the-negative-impacts-of-climate-change-in-northern-vanua-levu.

[123] Kiwa Initiative. Reforestation and coral reef restoration. https://kiwainitiative.org/en/projects/lamacca-ecosystem-restoration-and-conservation-project.

[124] Kiwa Initiative. Restoration of sea cucumbers wild stock / Women fisheries. https://kiwainitiative.org/en/projects/filters/cobenefits/biodiversity-conservation/restoration-of-sea-cucumber-wild-stock-to-improve-women-fisheries-project.

[125] Kiwa Initiative. UA HUKA / Protect ua huka, development of the ua huka biodiversity protection program. https://kiwainitiative.org/en/projects/filters/cobenefits/biodiversity-conservation/ua-huka.

[126] Knafo, S. E. *et al*. Sterilisation of hybrid Galapagos tortoises (*Geochelone nigra*) for island restoration. Part 1: endoscopic oophorectomy of females under ketamine‐medetomidine anaesthesia. *Veterinary Record* **168**, 47–47 (2011). 21257559 10.1136/vr.c6520

[127] Komugabe-Dixson, A. F., De Ville, N. S. E., Trundle, A. & McEvoy, D. Environmental change, urbanisation, and socio-ecological resilience in the Pacific: Community narratives from Port Vila, Vanuatu. *Ecosystem Services* **39**, 100973 (2019).

[128] Lee, D. & Jeon, S. W. Estimating Changes in Habitat Quality through Land-Use Predictions: Case Study of Roe Deer (*Capreolus pygargus tianschanicus*) in Jeju Island. *Sustainability* **12**, 10123 (2020).

[129] Lee, G.-A., Kim, J.-E. & Hong, S.-K. Historical, Geographical, and Biocultural Values of ‘Doksal’, Korean Stone Tidal Weirs. *JMIC* **12** (2023).

[130] Lezberg, A. L., Buresch, K., Neill, C. & Chase, T. Mechanical Land Clearing to Promote Establishment of Coastal Sandplain Grassland and Shrubland Communities. *Restoration Ecology* **14**, 220–232 (2006).

[131] Li, X. *et al*. Study on Stability and Ecological Restoration of Soil-Covered Rocky Slope of an Abandoned Mine on an Island in Rainy Regions. *Sustainability* **14**, 12959 (2022).

[132] Lin, Q. *et al*. Remotely Sensed Ecological Protection Redline and Security Pattern Construction: A Comparative Analysis of Pingtan (China) and Durban (South Africa). *Remote Sensing* **13**, 2865 (2021).

[133] Liu, J., Gong, X., Li, L., Chen, F. & Zhang, J. Innovative design and construction of the sponge city facilities in the Chaotou Park, Talent Island, Jiangmen, China. *Sustainable Cities and Society* **70**, 102906 (2021).

[134] Loh, R. K. & Daehler, C. C. Influence of Invasive Tree Kill Rates on Native and Invasive Plant Establishment in a Hawaiian Forest. *Restoration Ecology* **15**, 199–211 (2007).

[135] Losfeld, G., L’Huillier, L., Fogliani, B., Jaffré, T. & Grison, C. Mining in New Caledonia: environmental stakes and restoration opportunities. *Environ Sci Pollut Res* **22**, 5592–5607 (2015). 10.1007/s11356-014-3358-x 25065482

[136] Louzao, M., Arcos, J. M., Guijarro, B., Valls, M. & Oro, D. Seabird-trawling interactions: factors affecting species-specific to regional community utilisation of fisheries waste: Seabird-trawler interactions: species versus community. *Fisheries Oceanography* **20**, 263–277 (2011).

[137] Macreadie, P. I. *et al*. Seagrasses produce most of the soil blue carbon in three Maldivian islands. *Front. Mar. Sci.* **11**, 1359779 (2024).

[138] Mark, A. F., Baylis, G. T. S. & Dickinson, K. J. M. Monitoring the impacts of deer on vegetation condition of Secretary Island, Fiordland National Park, New Zealand: A clear case for deer control and ecological restoration. *Journal of the Royal Society of New Zealand* **21**, 43–54 (1991).

[139] Márquez, C., Gibbs, J. P., Carrión, V., Naranjo, S. & Llerena, A. Population Response of Giant Galápagos Tortoises to Feral Goat Removal. *Restoration Ecology* **21**, 181–185 (2013).

[140] Martinez Morales, R., Miura, T. & Idol, T. An assessment of Hawaiian dry forest condition with fine resolution remote sensing. *Forest Ecology and Management* **255**, 2524–2532 (2008).

[141] McEvoy, D. *et al*. Localized nature-based solutions for enhanced climate resilience and community wellbeing in urban informal settlements. *Climate and Development* **16**, 600–612 (2024).

[142] McNamara, K. E. *et al*. An assessment of community-based adaptation initiatives in the Pacific Islands. *Nat. Clim. Chang.* **10**, 628–639 (2020).

[143] Mihaere, S. *et al*. Centring localised indigenous concepts of wellbeing in urban nature-based solutions for climate change adaptation: case-studies from Aotearoa New Zealand and the Cook Islands. *Front. Environ. Sci.* **12**, 1278235 (2024).

[144] Mira, E. *et al*. Investigation of the Asexual Reproductive Characteristics of Native Species for Soil Bioengineering in the West Indies. *JTFS* **33**, 333–342 (2021).

[145] Mira, E. *et al*. The Conservation and Restoration of Riparian Forests along Caribbean Riverbanks Using Legume Trees. *Sustainability* **14**, 3709 (2022).

[146] Miskelly, C. M., Charteris, M. R. & Fraser, J. R. Successful translocation of Snares Island snipe (*Coenocorypha huegeli*) to replace the extinct South Island snipe (*C. iredalei*). *Notornis* **59**, 32–38 (2012).

[147] Montoya Maya, P. H., Smit, K. P., Burt, A. J. & Frias-Torres, S. Large-scale coral reef restoration could assist natural recovery in Seychelles, Indian Ocean. *NC* **16**, 1–17 (2016).

[148] Morris, J. T. & Staver, L. W. Elevation Changes in Restored Marshes at Poplar Island, Chesapeake Bay, MD: II. Modeling the Importance of Marsh Development Time. *Estuaries and Coasts* **47**, 1799–1813 (2024).

[149] Motamedi, S., Hashim, R., Zakaria, R., Song, K.-I. & Sofawi, B. Long-Term Assessment of an Innovative Mangrove Rehabilitation Project: Case Study on Carey Island, Malaysia. *The Scientific World Journal* **2014**, 1–12 (2014). 10.1155/2014/953830 PMC4109262 25097894

[150] Nappi, B. Twenty-five years of work at Poplar Island brings improved habitat, expanded use of dredged material. *World Dredging, Mining and Construction* **52**, 6–11 (2020).

[151] Nature4Climate. Guadeloupe Port Caraïbes: putting climate change at the heart of the port’s activity, (AIVP). https://nature4climate.org/nature-in-action/case-studies/.

[152] Nature4Climate. NIHT Topaiyo REDD+ Project, (NIHT Inc.). https://nature4climate.org/nature-in-action/case-studies/.

[153] Negoita, L., Gibbs, J. P. & Jaramillo Díaz, P. Cost‐effectiveness of water‐saving technologies for restoration of tropical dry forest: a case study from the Galapagos Islands, Ecuador. *Restoration Ecology* **30**, e13576 (2022).

[154] Nestlerode, J. A., Luckenbach, M. W. & O’Beirn, F. X. Settlement and Survival of the Oyster *Crassostrea virginica* on Created Oyster Reef Habitats in Chesapeake Bay. *Restoration Ecology* **15**, 273–283 (2007).

[155] NetworkNature. Blue Reef Project: Rebuilding of Marine Cavernous Boulder Reefs in Kattegat. https://networknature.eu/casestudy/26817.

[156] NetworkNature. Floating Wetland System on Utö. https://networknature.eu/casestudy/26786.

[157] NetworkNature. Urban Resilience on the Frontline of Climate Change. https://networknature.eu/casestudy/22860.

[158] Ng, K. *et al*. Multifunctional artificial reefs for small islands: An evaluation of amenity and opportunity for São Miguel Island, the Azores. *Progress in Physical Geography: Earth and Environment* **39**, 220–257 (2015).

[159] Nunn, P. D., Klöck, C. & Duvat, V. Seawalls as maladaptations along island coasts. *Ocean & Coastal Management* **205**, 105554 (2021).

[160] Olivé, I. *et al*. Contribution of the seagrass *Syringodium isoetifolium* to the metabolic functioning of a tropical reef lagoon. *Front. Mar. Sci.* **9**, 867986 (2022).

[161] Oliveira, N. M. & Hilker, F. M. Modelling Disease Introduction as Biological Control of Invasive Predators to Preserve Endangered Prey. *Bull. Math. Biol.* **72**, 444–468 (2010). 19787407 10.1007/s11538-009-9454-2

[162] Oppla. An assessment of green infrastructure and ecosystem services in the Valletta urban area: a case-study for sustainable urban planning. https://oppla.eu/casestudy/19309.

[163] Oppla. Assessing and mapping ecosystem services in the mosaic landscapes of the Maltese Islands. https://oppla.eu/casestudy/18249.

[164] Oppla. Assessing urban recreation ecosystem services through the use of geocache visitation and preference data: a case-study from an urbanised island environment. https://oppla.eu/casestudy/18461.

[165] Oppla. Blue Carbon in the Balearic Islands: Co-beneficiary management of seagrass ecosystems (Ibiza). https://oppla.eu/casestudy/17271.

[166] Oppla. Blue Carbon in the Balearic Islands: Co-beneficiary management of seagrass ecosystems (Mallorca). https://oppla.eu/casestudy/17271.

[167] Oppla. Blue Carbon in the Balearic Islands: Co-beneficiary management of seagrass ecosystems (Menorca). https://oppla.eu/casestudy/17271.

[168] Oppla. H2020 AQUACROSS Case Study 8 - Ecosystem-based solutions to solve sectoral conflicts on the path to sustainable development in the Faial-Pico Channel, Azores (Faial). https://oppla.eu/casestudy/18346.

[169] Oppla. H2020 AQUACROSS Case Study 8 - Ecosystem-based solutions to solve sectoral conflicts on the path to sustainable development in the Faial-Pico Channel, Azores (Pico). https://oppla.eu/casestudy/18346.

[170] Oppla. Healthy reefs for recreation, fisheries and flood protection on St. Eustatius. https://oppla.eu/casestudy/17279.

[171] Oppla. Integrated Urban Resilience Sector project in Tonga. https://oppla.eu/casestudy/24374.

[172] Oppla. Multifunctional Wetlands in Åland. https://oppla.eu/casestudy/27011.

[173] Oppla. Reunion Island Anchor Project / La Reunion Biocorridor. https://oppla.eu/casestudy/30248.

[174] Oppla. The First Large Land-Restoration Initiative in the Faroe Islands. https://oppla.eu/casestudy/26965.

[175] Oppla. The Nabben Multifunctional Wetland in Åland. https://oppla.eu/casestudy/28614.

[176] Orta-Ortiz, M. S. & Geneletti, D. Prioritizing urban nature-based solutions to support scaling-out strategies: A case study in Las Palmas de Gran Canaria. *Environmental Impact Assessment Review* **102**, 107158 (2023).

[177] PANORAMA. A lifebelt for Cousin Island Special Reserve MPA. https://panorama.solutions/en/solution/lifebelt-cousin-island-special-reserve-mpa.

[178] PANORAMA. Balancing Act: Managing Native Plants and Grazing Horses on Assateague Island. https://panorama.solutions/en/solution/balancing-act-managing-native-plants-and-grazing-horses-assateague-island.

[179] PANORAMA. Barcoding Galapagos: Recording and mitigating Covid-19 impacts using key-workers in eco-tourism. https://panorama.solutions/en/solution/barcoding-galapagos-recording-and-mitigating-covid-19-impacts-using-key-workers-eco.

[180] PANORAMA. Black coral conservation on the island of Cozumel. https://panorama.solutions/en/solution/black-coral-conservation-island-cozumel.

[181] PANORAMA. Co-producing deep sea science for a more equitable and inclusive future. https://panorama.solutions/en/solution/co-producing-deep-sea-science-more-equitable-and-inclusive-future.

[182] PANORAMA. Conservation Dogs & Drones for the protection of endangered sea turtles in Cabo Verde. https://panorama.solutions/en/solution/conservation-dogs-drones-protection-endangered-sea-turtles-cabo-verde.

[183] PANORAMA. Coral Gardening for Climate Change Adaptation in Vanuatu. https://panorama.solutions/en/solution/coral-gardening-climate-change-adaptation-vanuatu.

[184] PANORAMA. Creación del Fondo de Inversión Sostenible de la Reserva Marina de Galápagos. https://panorama.solutions/es/solution/creacion-del-fondo-de-inversion-sostenible-de-la-reserva-marina-de-galapagos.

[185] PANORAMA. Crowd funding for Marine Protected Area management. https://panorama.solutions/en/solution/crowd-funding-marine-protected-area-management.

[186] PANORAMA. Designation of Astola Island, Pakistan’s first Marine Protected Area. https://panorama.solutions/en/solution/designation-astola-island-pakistans-first-marine-protected-area.

[187] PANORAMA. Développement du réseau d’Aires marines éducatives. https://panorama.solutions/fr/solution/developpement-du-reseau-daires-marines-educatives.

[188] PANORAMA. Documenting the Life History Interviews of Robben Island Ex-Political Prisoners. https://panorama.solutions/en/solution/documenting-life-history-interviews-robben-island-ex-political-prisoners.

[189] PANORAMA. Empowering island communities: the use of cost-benefit analysis to support informed climate change adaptation decisions. https://panorama.solutions/en/solution/empowering-island-communities-use-cost-benefit-analysis-support-informed-climate-change.

[190] PANORAMA. Ensuring the Sustainability of Wastewater Operations in West End, Roatán. https://panorama.solutions/en/solution/ensuring-sustainability-wastewater-operations-west-end-roatan.

[191] PANORAMA. Estableciendo las bases para la cooperación transfronteriza en Planificación Espacial Marina en la Macaronesia europea. https://panorama.solutions/en/solution/estableciendo-las-bases-para-la-cooperacion-transfronteriza-en-planificacion-espacial.

[192] PANORAMA. Étude des échinodermes de Mayotte et sensibilisation. https://panorama.solutions/fr/solution/etude-des-echinodermes-de-mayotte-et-sensibilisation.

[193] PANORAMA. Évaluation de la distribution spatiale d’une espèce d’holothurie (*Holothuria fuscogilva*) dans le lagon de Vairao (Tahiti). https://panorama.solutions/fr/solution/evaluation-de-la-distribution-spatiale-dune-espece-dholothurie-holothuria-fuscogilva-dans.

[194] PANORAMA. Funding the Aldabra Clean Up Project through Corporate sponsorship and Crowdfunding. https://panorama.solutions/en/solution/funding-aldabra-clean-project-through-corporate-sponsorship-and-crowdfunding.

[195] PANORAMA. Guanaja Mangrove Restoration Project. https://panorama.solutions/en/solution/guanaja-mangrove-restoration-project.

[196] PANORAMA. Implementación del “Proyecto Estudio, Registry y Monitoreo de Sitios Arqueológicos” Indicador de Sustentabilidad del Recurso Arqueológico Patrimonial en Rapa Nui. https://panorama.solutions/en/solution/implementacion-del-proyecto-estudio-registro-y-monitoreo-de-sitios-arqueologicos-indicador.

[197] PANORAMA. Improved capacity for Conservation Action and Sustainable Management: Data collection to inform biodiversity conservation, wildlife protection and general area surveillance programmes within the PSEPA. https://panorama.solutions/en/solution/improved-capacity-conservation-action-and-sustainable-management-data-collection-inform.

[198] PANORAMA. Improving trails and visitor experiences in the Peaks National Park, St Helena Island. https://panorama.solutions/en/solution/improving-trails-and-visitor-experiences-peaks-national-park-st-helena-island.

[199] PANORAMA. Integrating seagrass into the Seychellois Creole language. https://panorama.solutions/en/solution/integrating-seagrass-seychellois-creole-language.

[200] PANORAMA. Intégrer les cultivateurs de Vanille dans la conservation de la biodiversité des forêts tropicales. https://panorama.solutions/fr/solution/integrer-les-cultivateurs-de-vanille-dans-la-conservation-de-la-biodiversite-des-forets.

[201] PANORAMA. Isn’t there an App for that? Smartphone Apps in marine resource management. https://panorama.solutions/en/solution/isnt-there-app-smartphone-apps-marine-resource-management.

[202] PANORAMA. Lands of Priolo: Integrated management to save a bird, recover natural habitats and promote sustainability. https://panorama.solutions/en/solution/lands-priolo-integrated-management-save-bird-recover-natural-habitats-and-promote.

[203] PANORAMA. Larval propagation to assist the recovery and resilience of Bonaire coral populations in the face of new diseases and environmental changes. https://panorama.solutions/en/solution/larval-propagation-assist-recovery-and-resilience-bonaire-coral-populations-face-new.

[204] PANORAMA. Lauru Ridges to Reefs Protected Area Network (Lauru PAN). https://panorama.solutions/en/solution/lauru-ridges-reefs-protected-area-network-lauru-pan.

[205] PANORAMA. Mafia Island Marine Park: a success story of inclusive governance. https://panorama.solutions/en/solution/mafia-island-marine-park-success-story-inclusive-governance.

[206] PANORAMA. Mangrove Restoration Bonaire. https://panorama.solutions/en/solution/mangrove-restoration-bonaire.

[207] PANORAMA. Maristanis: an integrated coastal and wetlands management. https://panorama.solutions/en/solution/maristanis-integrated-coastal-and-wetlands-management.

[208] PANORAMA. Marking a No-take Area in the Community of Navakavu. https://panorama.solutions/en/solution/marking-no-take-area-community-navakavu.

[209] PANORAMA. Participatory 3D Mapping for Land Use Planning and Climate Change Adaptation. https://panorama.solutions/en/solution/participatory-3d-mapping-land-use-planning-and-climate-change-adaptation.

[210] PANORAMA. Protected Area management on private islands: innovate finance examples from Denis and North Islands, Seychelles (Denis Island). https://panorama.solutions/en/solution/protected-area-management-private-islands-innovate-finance-examples-denis-and-north.

[211] PANORAMA. Protected Area management on private islands: innovate finance examples from Denis and North Islands, Seychelles (North Island). https://panorama.solutions/en/solution/protected-area-management-private-islands-innovate-finance-examples-denis-and-north.

[212] PANORAMA. Protecting Chumbe Island Nature Reserve from increased poaching threats due to COVID-19 pandemic, through re-assignment of local rangers, skills development of youth fishers, and sustaining its conservation activities. https://panorama.solutions/en/solution/protecting-chumbe-island-nature-reserve-increased-poaching-threats-due-covid-19-pandemic.

[213] PANORAMA. QBook – A QGIS Cookbook for the Pacific GIS Community. https://panorama.solutions/en/solution/qbook-qgis-cookbook-pacific-gis-community.

[214] PANORAMA. Recovering the administration of ancestral land: the establishment of the Indigenous Community Ma’u Henua, stewards of Rapa Nui National Park, Chile. https://panorama.solutions/en/solution/recovering-administration-ancestral-land-establishment-indigenous-community-mau-henua.

[215] PANORAMA. Renforcer la capacité des populations locales à la production de plants pour la restauration de sites dégradés. https://panorama.solutions/fr/solution/renforcer-la-capacite-des-populations-locales-la-production-de-plants-pour-la-restauration.

[216] PANORAMA. Restoration of Kamaka Island, a sanctuary for Gambier biodiversity. https://panorama.solutions/en/solution/restoration-kamaka-island-sanctuary-gambier-biodiversity.

[217] PANORAMA. Safe Island for Seabirds (Corvo). https://panorama.solutions/en/solution/safe-island-seabirds.

[218] PANORAMA. Safe Island for Seabirds (Vila Franca do Campo). https://panorama.solutions/en/solution/safe-island-seabirds.

[219] PANORAMA. Safeguarding the Underwater Cultural Heritage of Stone Tidal Weirs on the Earth. https://panorama.solutions/en/solution/safeguarding-underwater-cultural-heritage-stone-tidal-weirs-earth.

[220] PANORAMA. Seabird Habitat Restoration Program - Montague Island Nature Reserve. https://panorama.solutions/en/solution/seabird-habitat-restoration-program-montague-island-nature-reserve.

[221] PANORAMA. Seaweed farming in Zanzibar: addressing the common challenge of aquaculture and marine conservation (Pemba Island). https://panorama.solutions/en/solution/seaweed-farming-zanzibar-addressing-common-challenge-aquaculture-and-marine-conservation.

[222] PANORAMA. Seaweed farming in Zanzibar: addressing the common challenge of aquaculture and marine conservation (Unguja Island). https://panorama.solutions/en/solution/seaweed-farming-zanzibar-addressing-common-challenge-aquaculture-and-marine-conservation.

[223] PANORAMA. Shelters for archaeological sites: protecting heritage and enhancing visitors’ experience. https://panorama.solutions/en/solution/shelters-archaeological-sites-protecting-heritage-and-enhancing-visitors-experience.

[224] PANORAMA. Solar street furniture dedicated to the collection of recyclable packaging. https://panorama.solutions/en/solution/solar-street-furniture-dedicated-collection-recyclable-packaging.

[225] PANORAMA. Soqotra Heritage Project: building local capacities for the protection of the unique cultural and natural heritage of Soqotra. https://panorama.solutions/en/solution/soqotra-heritage-project-building-local-capacities-protection-unique-cultural-and-natural.

[226] PANORAMA. Stimulating sustainable financing for long-term coral reef conservation in the Turks and Caicos Islands (Providenciales). https://panorama.solutions/en/solution/stimulating-sustainable-financing-long-term-coral-reef-conservation-turks-and-caicos.

[227] PANORAMA. Stimulating sustainable financing for long-term coral reef conservation in the Turks and Caicos Islands (West Caicos). https://panorama.solutions/en/solution/stimulating-sustainable-financing-long-term-coral-reef-conservation-turks-and-caicos.

[228] PANORAMA. Supporting transboundary dialogue on Marine Spatial Planning towards a sustainable blue economy for the Western Mediterranean. https://panorama.solutions/en/solution/supporting-transboundary-dialogue-marine-spatial-planning-towards-sustainable-blue-economy.

[229] PANORAMA. Testing new low-tech ecological restoration techniques within tribal communities in New Caledonia. https://panorama.solutions/en/solution/testing-new-low-tech-ecological-restoration-techniques-within-tribal-communities-new.

[230] PANORAMA. The conservation and protection of seabirds in Kiritimati Island. https://panorama.solutions/en/solution/conservation-and-protection-seabirds-kiritimati-island.

[231] PANORAMA. The Palau National Marine Sanctuary: Protecting a nation’s entire marine territory to ensure sustainable development, enhance food security, boost tourism and enrich biodiversity conservation. https://panorama.solutions/en/solution/palau-national-marine-sanctuary-protecting-nations-entire-marine-territory-ensure.

[232] PANORAMA. Toward conservation of coastal sharks in the French West Indies. https://panorama.solutions/fr/solution/toward-conservation-coastal-sharks-french-west-indies.

[233] PANORAMA. Transitioning to Low Carbon Sea Transport in the Marshall Islands. https://panorama.solutions/en/solution/transitioning-low-carbon-sea-transport-marshall-islands.

[234] PANORAMA. Trawangan Innovation and Education towards Zero Waste. https://panorama.solutions/en/solution/waste-management-very-touristic-area-gili-trawangan-innovation-and-education-towards-zero.

[235] PANORAMA. Using the IMET to identify priorities for management in two protected areas in Boa Vista, Cabo Verde. https://panorama.solutions/en/solution/using-imet-identify-priorities-management-two-protected-areas-boa-vista-cabo-verde.

[236] Park, H. & Higgs, E. A criteria and indicators monitoring framework for food forestry embedded in the principles of ecological restoration. *Environ Monit Assess* **190**, 113 (2018). 29396659 10.1007/s10661-018-6494-9

[237] Pearson, J., McNamara, K. E. & Nunn, P. D. iTaukei Ways of Knowing and Managing Mangroves for Ecosystem-Based Adaptation. in *Managing Climate Change Adaptation in the Pacific Region* (ed. Leal Filho, W.) 105–127, 10.1007/978-3-030-40552-6_6 (Springer International Publishing, Cham, 2020).

[238] Pedersen Zari, M. *et al*. Devising urban ecosystem-based adaptation (EbA) projects with developing nations: A case study of Port Vila, Vanuatu. *Ocean & Coastal Management* **184**, 105037 (2020).

[239] Pedersen Zari, M., Kiddle, G. L., Blaschke, P., Gawler, S. & Loubser, D. Utilising nature-based solutions to increase resilience in Pacific Ocean Cities. *Ecosystem Services* **38**, 100968 (2019).

[240] Pejchar, L., Gallo, T., Hooten, M. B. & Daily, G. C. Predicting effects of large‐scale reforestation on native and exotic birds. *Diversity and Distributions* **24**, 811–819 (2018).

[241] Penn, T. & Tomasi, T. Calculating Resource Restoration for an Oil Discharge in Lake Barre, Louisiana, USA. *Environmental Management* **29**, 691–702 (2002). 12180182 10.1007/s00267-001-0059-2

[242] Pesendorfer, M. B. *et al*. Oak habitat recovery on California’s largest islands: Scenarios for the role of corvid seed dispersal. *Journal of Applied Ecology* **55**, 1185–1194 (2018).

[243] Plunkett, E., Negoita, L., Sevilla, C., Velasco, N. & Jaramillo Díaz, P. Enhancing restoration success of rare plants in an arid-tropical climate through water-saving technologies: a case study of *Scalesia affinis* ssp. *brachyloba* in the Galapagos Islands. *PeerJ* **11**, e16367 (2023). 38077418 10.7717/peerj.16367 PMC10710167

[244] Poff, M., Bass, A., Sweeney, R., Bahlinger, K. & Chatellier, M. Feasibility Study Analysis for Riverine Sand Mining/Scofield Island Restoration. *Journal of Coastal Research* **59**, 235–245 (2011).

[245] Quinzin, M. C. *et al*. Genetically informed captive breeding of hybrids of an extinct species of Galapagos tortoise. *Conservation Biology* **33**, 1404–1414 (2019). 30901116 10.1111/cobi.13319

[246] Ray, G. J. & Brown, B. J. Restoring Caribbean Dry Forests: Evaluation of Tree Propagation Techniques. *Restoration Ecology* **3**, 86–94 (1995).

[247] Reguero, B. G., Beck, M. W., Agostini, V. N., Kramer, P. & Hancock, B. Coral reefs for coastal protection: A new methodological approach and engineering case study in Grenada. *Journal of Environmental Management* **210**, 146–161 (2018). 29339333 10.1016/j.jenvman.2018.01.024

[248] Rivillas-Ospina, G. *et al*. Alternatives for Recovering the Ecosystem Services and Resilience of the Salamanca Island Natural Park, Colombia. *Water* **12**, 1513 (2020).

[249] Rodríguez, B., Rodríguez, A., Lorenzo, J. A. & Martínez, J. M. Exotic tree plantations as alternative breeding habitat for an endemic avian predator. *Journal of Avian Biology* **52**, jav.02527 (2021).

[250] Roffler, G. H., Eriksson, C. E., Allen, J. M. & Levi, T. Recovery of a marine keystone predator transforms terrestrial predator–prey dynamics. *Proc. Natl. Acad. Sci. USA.* **120**, e2209037120 (2023). 36689656 10.1073/pnas.2209037120 PMC9945949

[251] Roosenburg, W. M. *et al*. Nesting Habitat Creation Enhances Recruitment in a Predator‐Free Environment: *Malaclemys* Nesting at the Paul S. Sarbanes Ecosystem Restoration Project. *Restoration Ecology* **22**, 815–823 (2014).

[252] Sahin, O. *et al*. Assessing how ecosystem-based adaptations to climate change influence community wellbeing: a Vanuatu case study. *Reg Environ Change* **21**, 90 (2021).

[253] Shi, H. *et al*. A model to assess fundamental and realized carrying capacities of island ecosystem: A case study in the southern Miaodao Archipelago of China. *Acta Oceanol. Sin.* **35**, 56–67 (2016).

[254] Slinger, J. H. & Kothuis, B. B. A specific transdisciplinary co-design workshop model to teach a multiple perspective problem approach for integrated nature-based design. in *Coastal Flood Risk Reduction* 377–395, 10.1016/B978-0-323-85251-7.00028-7 (Elsevier, 2022).

[255] Soanes, L. M. *et al*. Reducing the vulnerability of coastal communities in the Caribbean through sustainable mangrove management. *Ocean & Coastal Management* **210**, 105702 (2021).

[256] Stankovic, M. *et al*. Quantification of blue carbon in seagrass ecosystems of Southeast Asia and their potential for climate change mitigation. *Science of The Total Environment* **783**, 146858 (2021). 34088119 10.1016/j.scitotenv.2021.146858

[257] Staver, L. W. *et al*. Elevation Changes in Restored Marshes at Poplar Island, Chesapeake Bay, MD: I. Trends and Drivers of Spatial Variability. *Estuaries and Coasts* **47**, 1784–1798 (2024).

[258] Summers, R., Masukawa, J. & Hartman, B. D. The Influence of Slope on Vegetation Recovery Following Nonnative Grazer Removal on Santa Rosa Island, California. *Western North American Naturalist* **78**, 787 (2018).

[259] Tassin, J., Rivière, J. & Clergeau, P. Reproductive versus Vegetative Recruitment of the Invasive Tree *Schinus terebenthifolius*: Implications for Restoration on Reunion Island. *Restoration Ecology* **15**, 412–419 (2007).

[260] Taylor, C. N., Russell, J. C. & Russell, K. J. A strategic social impact assessment for Predator-Free Rakiura, New Zealand, with a human–ecological approach. *Socio Ecol Pract Res* **2**, 161–174 (2020).

[261] Thomaidi, V., Petousi, I., Kotsia, D., Kalogerakis, N. & Fountoulakis, M. S. Use of green roofs for greywater treatment: Role of substrate, depth, plants, and recirculation. *Science of The Total Environment* **807**, 151004 (2022). 34666091 10.1016/j.scitotenv.2021.151004

[262] Towns, D. R. Korapuki Island as a case study for restoration of insular ecosystems in New Zealand. *Journal of Biogeography* **29**, 593–607 (2002).

[263] Travers, T., Lea, M., Alderman, R., Terauds, A. & Shaw, J. Bottom‐up effect of eradications: The unintended consequences for top‐order predators when eradicating invasive prey. *Journal of Applied Ecology* **58**, 801–811 (2021).

[264] Urban Nature Atlas. Ecosystem-based Adaptation to Climate Change. https://una.city/NbS/victoria/ecosystem-based-adaptation-climate-change.

[265] Urban Nature Atlas. Green elements, the Poligono Levante reurbanization. https://una.city/NbS/palma-de-mallorca/green-elements-poligono-levante-reurbanization.

[266] Urban Nature Atlas. Interior Vertical Garden Santa Ponca. https://una.city/NbS/palma-de-mallorca/interior-vertical-garden-santa-ponca.

[267] Urban Nature Atlas. Krekovic Park green rehabilitation. https://una.city/NbS/palma-de-mallorca/krekovic-park-green-rehabilitation.

[268] Urban Nature Atlas. Resilient Islands Project. https://una.city/NbS/grenville-bay/resilient-islands-project.

[269] Urban Nature Atlas. Sea’s Corridor. https://una.city/NbS/palma-de-mallorca/seas-corridor.

[270] Urban Nature Atlas. Siargao It Up: Mangrove Conservation. https://una.city/NbS/del-carmen/siargao-it-mangrove-conservation.

[271] Urban Nature Atlas. Tagabe Riparian Corridor Regeneration Project. https://una.city/NbS/port-vila/tagabe-riparian-corridor-regeneration-project.

[272] Urban Nature Atlas. Tracks Park Corridor. https://una.city/NbS/palma-de-mallorca/tracks-park-corridor.

[273] Urban Nature Atlas. Urban Forest. https://una.city/NbS/palma-de-mallorca/urban-forest.

[274] Urban Nature Atlas. Vertical Garden. https://una.city/NbS/palma-de-mallorca/vertical-garden.

[275] Urban Nature Atlas. Vias Park (Green Pathways). https://una.city/NbS/palma-de-mallorca/vias-park-green-pathways.

[276] Van Leeuwen, C. H. A. *et al*. Enhancing ecological integrity while preserving ecosystem services: Constructing soft‐sediment islands in a shallow lake. *Ecol Sol and Evidence* **2**, e12098 (2021).

[277] Walters, B. B. People and mangroves in the Philippines: fifty years of coastal environmental change. *Envir. Conserv.* **30**, 293–303 (2003).

[278] Wang, L. *et al*. Biological soil crust elicits microbial community and extracellular polymeric substances restructuring to reduce the soil erosion on tropical island, South China Sea. *Marine Environmental Research* **197**, 106449 (2024). 38492504 10.1016/j.marenvres.2024.106449

[279] Weijerman, M. *et al*. Towards an ecosystem-based approach of Guam’s coral reefs: The human dimension. *Marine Policy* **63**, 8–17 (2016).

[280] Weller, S. G. *et al*. Alien Plant Invasions, Introduced Ungulates, and Alternative States in a Mesic Forest in Hawaii. *Restoration Ecology* **19**, 671–680 (2011).

[281] Wen, X., Ming, Y., Gao, Y. & Hu, X. Dynamic Monitoring and Analysis of Ecological Quality of Pingtan Comprehensive Experimental Zone, a New Type of Sea Island City, Based on RSEI. *Sustainability* **12**, 21 (2019).

[282] Wilkinson, S. R., Naeth, M. A. & Schmiegelow, F. K. A. Tropical Forest Restoration within Galapagos National Park: Application of a State-transition Model. *Ecology and Society* **10** (2005).

[283] WOCAT. Afforestation. https://qcat.wocat.net/en/wocat/technologies/view/technologies_1523/.

[284] WOCAT. Aloe Vera Living Barriers. https://qcat.wocat.net/en/wocat/technologies/view/technologies_1334/.

[285] WOCAT. Application of biological agents to increase crop resistance to salinity. https://qcat.wocat.net/en/wocat/technologies/view/technologies_1281/.

[286] WOCAT. Barreiras Vivas de Leucaena / Leucanea Living Barriers. https://qcat.wocat.net/en/wocat/technologies/view/technologies_1574/.

[287] WOCAT. Crop rotation for green manuring in greenhouse. https://qcat.wocat.net/en/wocat/technologies/view/technologies_1246/.

[288] WOCAT. Establishment of intensive grazing areas on low productive slopes. https://qcat.wocat.net/en/wocat/technologies/view/technologies_2900/.

[289] WOCAT. Land terracing in olive groves. https://qcat.wocat.net/en/wocat/technologies/view/technologies_1512/.

[290] WOCAT. Mangroves as Buffer against Natural Hazards. https://qcat.wocat.net/en/wocat/technologies/view/technologies_578/.

[291] WOCAT. Olive groves under no-tillage operations. https://qcat.wocat.net/en/wocat/technologies/view/technologies_1035/.

[292] WOCAT. Reforced terraces for stone walls [Cape Verde]. https://qcat.wocat.net/en/wocat/technologies/view/technologies_1573/.

[293] WOCAT. Soil erosion control by ridges. https://qcat.wocat.net/en/wocat/technologies/view/technologies_2922/.

[294] WOCAT. Training, information and awareness raising. https://qcat.wocat.net/en/wocat/approaches/view/approaches_2420/.

[295] WOCAT. Water and soil conservation by using rock fragments. https://qcat.wocat.net/en/wocat/technologies/view/technologies_2911/.

[296] WOCAT. WINDBREAKS. https://qcat.wocat.net/en/wocat/technologies/view/technologies_1421/.

[297] Woolsey, J., Hanna, C., Mceachern, K., Anderson, S. & Hartman, B. D. Regeneration and Expansion of *Quercus tomentella* (Island Oak) Groves on Santa Rosa Island. *Western North American Naturalist* **78**, 758 (2018).

[298] Wu, L., Ouyang, Y., Cai, L., Dai, J. & Wu, Y. Ecological restoration approaches for degraded muddy coasts: Recommendations and practice. *Ecological Indicators* **149**, 110182 (2023).

[299] Xia, J. *et al*. The effect of two types of grid transplantation on coral growth and the *in-situ* ecological restoration in a fragmented reef of the South China Sea. *Ecological Engineering* **177**, 106558 (2022).

[300] Yelenik, S. G. & Levine, J. M. Native shrub reestablishment in exotic annual grasslands: Do ecosystem processes recover? *Ecological Applications* **20**, 716–727 (2010). 20437958 10.1890/08-2365.1

[301] Yelenik, S. G. & Levine, J. M. Processes Limiting Native Shrub Recovery in Exotic Grasslands after Non‐Native Herbivore Removal. *Restoration Ecology* **18**, 418–425 (2010).

[302] Zaimes, G. N. *et al*. Targeted placement of soil erosion prevention works after wildfires. *IOP Conf. Ser.: Earth Environ. Sci.* **612**, 012050 (2020).

[303] Zheng, X., Li, Y., Liang, J., Lin, R. & Wang, D. Performance of ecological restoration in an impaired coral reef in the Wuzhizhou Island, Sanya, China. *J. Ocean. Limnol.* **39**, 135–147 (2021).

[304] Zhu, T. R., Litton, C. M., Giardina, C. P. & Trauernicht, C. Moisture availability and ecological restoration limit fine fuels and modelled wildfire intensity following non‐native ungulate removal in Hawaii. *Journal of Applied Ecology* **58**, 2207–2219 (2021).

[305] Deutsche Gesellschaft für Internationale Zusammenarbeit (GIZ). *PANORAMA Solutions for a Healthy Planet* https://panorama.solutions/en.

[306] Oppla Consortium. *Oppla* https://oppla.eu/.

[307] Physi Solutions. *Urban Nature Atlas* https://una.city/.

[308] N4C Secretariat, The Nature Conservancy (UK). *Nature4Climate* https://nature4climate.org/.

[309] NetworkNature Consortium. *NetworkNature* https://networknature.eu/.

[310] The Kiwa Initiative Team. *Kiwa Initiative* https://kiwainitiative.org/en/.

[311] The Equator Initiative Team. *Equator Initiative* https://www.equatorinitiative.org/.

[312] WOCAT Consortium. *WOCAT* https://wocat.net/en/.

